# Interparental Conflict and Adolescent Peer Bullying Victimization: A Longitudinal Moderated Mediation Model of Depressive Symptoms and Teacher–Student Relationships

**DOI:** 10.3390/healthcare14131844

**Published:** 2026-06-25

**Authors:** Chang Wei, Sitian Ma, Xiaojing An, Wei Zhang

**Affiliations:** 1 School of Arts and Sciences, Guangzhou Maritime University, Guangzhou 510725, China; 2 School of Psychology, South China Normal University, Guangzhou 510631, China; 3 School of Teacher Education, Xingtai University, Xingtai 054000, China

**Keywords:** adolescents, interparental conflict, depressive symptoms, peer bullying victimization, teacher–student relationships

## Abstract

**Highlights:**

**What are the main findings?**

- First, interparental conflict was positively associated with adolescent peer bullying victimization six months later, and depressive symptoms mediated this longitudinal association.
- Second, teacher–student relationships moderated both the association between interparental conflict and depressive symptoms, and the association between depressive symptoms and peer bullying victimization.

**What are the implications of the main findings?**

- First, interventions targeting adolescent depressive symptoms may help reduce peer bullying victimization, especially among youth exposed to interparental conflict.
- Second, school-based interventions to improve teacher–student relationships can buffer the negative impact of interparental conflict on depressive symptoms and weaken the link between depressive symptoms and bullying victimization.

**Abstract:**

**Background/Objectives**: Interparental conflict increases adolescents’ risk of peer bullying victimization, yet its underlying mechanisms remain underexplored. This study sought to examine whether depressive symptoms mediate the longitudinal relationship between interparental conflict and peer bullying victimization among adolescents, and whether teacher–student relationships moderate this mediating pathway. **Methods**: A two-wave longitudinal design spaced six months apart was adopted, with 759 Chinese adolescents participating across both waves. Interparental conflict, depressive symptoms, teacher–student relationships, and peer bullying victimization were measured using validated scales. Gender, age, and Wave 1 depressive symptoms and peer bullying victimization were controlled. Data were analyzed using SPSS PROCESS macro (Models 4 and 58). **Results**: Interparental conflict was positively associated with peer bullying victimization six months later (*r* = 0.14, *p* < 0.001). Depressive symptoms significantly mediated this relationship (indirect effect = 0.03, 95% CI = [0.01, 0.05]). Teacher–student relationships significantly moderated both the path from interparental conflict to depressive symptoms (*b* = −0.08, *p* < 0.05) and the path from depressive symptoms to peer bullying victimization (*b* = −0.16, *p* < 0.01). For adolescents with low teacher–student relationships, interparental conflict was positively associated with depressive symptoms (*b* = 0.10, *p* < 0.001), and depressive symptoms were positively associated with peer bullying victimization (*b* = 0.45, *p* < 0.001). For those with high teacher–student relationships, interparental conflict was not significantly associated with depressive symptoms (*b* = 0.01, *p* > 0.05), while depressive symptoms remained significantly but weakly associated with peer bullying victimization (*b* = 0.26, *p* < 0.001). **Conclusions**: Positive teacher–student relationships buffer the indirect association linking interparental conflict to peer bullying victimization via depressive symptoms. This occurs mainly by attenuating the association between interparental conflict and depressive symptoms to non-significance and lowering the magnitude of the association between depressive symptoms and peer bullying victimization. Higher-quality teacher–student relationships may weaken the correlational pathway connecting family conflict to peer bullying victimization.

## 1. Introduction

Peer bullying victimization refers to an individual’s experience of suffering attacks from peers in physical, verbal, property, and interpersonal relationship dimensions [1]. Peer bullying victimization is a relatively common phenomenon among adolescents. A recent study (with a sample of 2175 adolescents) showed that 43.0% of participants reported having experienced peer bullying, and 11.9% reported chronic peer bullying victimization [2]. Moreover, peer bullying victimization among adolescents is closely related to their future engagement in non-suicidal self-injury and suicidal behavior [3,4]. Therefore, elucidating the risk factors contributing to and protective factors buffering against adolescent peer bullying victimization is critical for informing evidence-based prevention and intervention.

Peer bullying victimization is influenced by multiple factors across individual, family, and school contexts. Previous research has identified numerous influencing factors, including individual characteristics (e.g., depressive symptoms) [5], family factors (e.g., interparental conflict) [6], and school factors (e.g., teacher–student relationships) [7]. However, most previous studies have focused on the independent effects of these factors, and how they interact to jointly predict peer bullying victimization remains to be further explored. Therefore, the present study integrates interparental conflict (a distal family risk factor), depressive symptoms (a proximal individual risk factor), and teacher–student relationships (a school protective factor) to construct a moderated mediation model, examining how interparental conflict affects peer bullying victimization through depressive symptoms and how teacher–student relationships moderate this mediating pathway.

### 1.1. Interparental Conflict and Peer Bullying Victimization

Research shows that family factors are pivotal to the occurrence of bullying behavior among adolescents [8], with interparental conflict being a significant risk factor for such behavior [9]. Interparental conflict refers to verbal or physical aggression and disputes between spouses arising from disagreements [10]. In this study, interparental conflict is conceptualized as a unidimensional construct, measured by a composite score of frequency, intensity, and resolution. The bioecological model of human development posits that the dynamic interaction between biological factors and ecological environmental factors influences individual development [11]. According to this model, negative interactions within the family environment, such as interparental conflict, may exert direct adverse effects on adolescents’ development and raise their risk of being bullied by peers. Empirical studies have consistently found a significant positive correlation between interparental conflict and peer bullying victimization among adolescents. For example, Ye [6] found in a study of 551 adolescents that interparental conflict was significantly and positively associated with peer bullying victimization.

Nevertheless, the specific mechanisms through which interparental conflict predicts bullying victimization among adolescents have not been fully elucidated. The mediating effect of depressive symptoms and the protective moderating role of teacher–student relationships remain to be further explored. Accordingly, the present study examines the mediating effect of depressive symptoms in the association between interparental conflict and bullying victimization, as well as the moderating effect of teacher–student relationships on this indirect pathway.

### 1.2. Depressive Symptoms as a Potential Mediator

The link between interparental conflict and adolescent bullying victimization may be indirect, with negative emotions (such as depression symptoms) potentially playing a key mediating role. Firstly, interparental conflict is a significant risk factor for the emergence of depressive symptoms among adolescents. The emotional security theory emphasizes that destructive conflicts between parents undermine adolescents’ emotional security and increase their risk of maladjustment [12]. According to this theory, interparental conflict may erode adolescents’ emotional security, thereby triggering depressive emotions. Empirical research has also confirmed that interparental conflict is a significant predictor of future depressive symptoms among adolescents [13]. Secondly, the symptom-driven model emphasizes that individuals’ psychological symptoms are the cause, rather than the consequence, of subsequent interpersonal difficulties [14,15,16]. According to this model, depressive emotions increase adolescents’ risk of being bullied in peer interactions. Relevant longitudinal studies have also shown that depressive symptoms predict later peer bullying victimization among adolescents [5,17]. Thirdly, previous studies have confirmed that depressive symptoms play a mediating role between family risk factors and adolescent bullying victimization. In a study involving 1259 adolescents, Dong and Zhang [18] reported that depressive symptoms served as a mediator in the relationship between family cumulative risk and bullying victimization.

### 1.3. Teacher–Student Relationships as a Moderator

Prior research has established that positive teacher–student relationships serve as a critical protective factor against peer bullying victimization among adolescents [7,19]. For example, in a longitudinal study of 930 early adolescents, ten Bokkel et al. [7] found that positive teacher–student relationships were significantly negatively associated with peer bullying victimization. The stress-buffering model emphasizes that social support plays a protective role when individuals face stress, attenuating the association between stressors and negative outcomes [20,21]. According to this model, positive teacher–student relationships, as an important source of social support, buffer the link between the stressor of interparental conflict and adolescent adjustment difficulties (e.g., depressive symptoms and peer bullying victimization). This perspective is corroborated by empirical research indicating that teacher–student relationships significantly moderate the relationship between stressful events and adverse developmental outcomes among adolescents [22,23]. For example, a study by Sun et al. [22] involving 1176 adolescents showed that teacher–student relationships significantly moderated the association between parental psychological control and problematic smartphone use. Similarly, Xie et al. [23] found that teacher–student relationships weakened the effect of perceived discrimination on loneliness among left-behind junior high school students whose mothers worked outside the home.

### 1.4. The Present Study

However, existing studies have not sufficiently explored the potential mechanisms underlying the association between interparental conflict and peer bullying victimization. Accordingly, the present study constructed a moderated mediation model using survey data from Chinese adolescents to examine the mediating effect of depressive symptoms and the moderating effect of teacher–student relationships on this mediating pathway. Specifically, this study tested the following hypotheses:

**Hypothesis** **1.** *Interparental conflict is positively associated with peer bullying victimization among adolescents.*

**Hypothesis** **2.** *Depressive symptoms mediate the positive relationship between interparental conflict and peer bullying victimization.*

**Hypothesis** **3.** *Teacher–student relationships moderate the indirect effect of interparental conflict on peer bullying victimization via depressive symptoms.*

## 2. Materials and Methods

### 2.1. Research Design

This study utilized a two-wave longitudinal design with a 6-month interval. Data were gathered from two ordinary junior high schools in China. The study aimed to examine the effect of interparental conflict on peer bullying victimization, as well as the mediating role of depressive symptoms and the moderating role of teacher–student relationships. The study obtained ethical approval from the Academic Committee of the researchers’ affiliated university. Prior to the survey, informed consent was obtained from the parents or legal guardians of all participating adolescents, as well as assent from the adolescents themselves.

In this study, interparental conflict (the independent variable) was measured only at Wave 1; depressive symptoms (the mediator) and peer bullying victimization (the dependent variable) were measured at both Wave 1 and Wave 2 (with Wave 1 values entered as control variables); teacher–student relationships (the moderator) were measured only at Wave 2. This design was based on the following considerations: (a) for the second half of the pathway (Wave 2 depressive symptoms → Wave 2 bullying victimization), measuring the moderator concurrently with the mediator and outcome variables is appropriate for testing this contemporaneous moderating effect; (b) for the first half of the pathway (Wave 1 interparental conflict → Wave 2 depressive symptoms), previous longitudinal research has shown that teacher–student relationships are relatively stable over time, with modest within-year fluctuations [24]. Therefore, teacher–student relationships measured at Wave 2 can, on the whole, reflect the environmental support available to adolescents during the interval from Wave 1 to Wave 2.

### 2.2. Study Setting and Participant Recruitment

This study was conducted in November 2024 (Wave 1) and April 2025 (Wave 2) in two ordinary public junior high schools located in China. A total of 771 students participated in Wave 1, and 759 completed Wave 2, with 12 participants lost to follow-up (attrition rate: 1.6%). The final analytical sample only retained participants who completed both waves, totaling 759 individuals. We compared retained participants and those who dropped out on key variables, and found no significant differences (ps > 0.05).

A cluster sampling method was adopted, with classes as the sampling units, and adolescents in the 7th and 8th grades of junior high school were selected as participants. Inclusion criteria: All students in the participating classes were invited to enroll, provided that they met the following conditions: (a) parental/guardian informed consent was obtained; (b) personal assent was provided; (c) from two-parent families; and (d) they were not absent due to leave requests on the survey days.

Survey procedures: The survey was conducted in regular classrooms by trained university teachers. After reading out standardized instructions, students completed paper-based questionnaires independently. The questionnaires were collected on site. All data were kept strictly confidential.

### 2.3. Measures

#### 2.3.1. Interparental Conflict (Independent Variable)

Interparental conflict was measured using the Conflict Properties Scale, which is a subscale extracted from The Children’s Perception of Interparental Conflict Scale (revised by Chi & Xin [25] and originally developed by Grych et al. [26]). Previous researchers [27,28] have typically used this subscale to assess the level of interparental conflict perceived by adolescents. This subscale includes three factors (i.e., conflict frequency, conflict intensity, and conflict resolution) and consists of 19 items (e.g., “I often see my mom and dad arguing”). Each item is rated on a 4-point Likert scale ranging from 1 (strongly disagree) to 4 (strongly agree). Higher mean scores indicate higher levels of interparental conflict. Cronbach’s alpha was 0.93 at Wave 1.

#### 2.3.2. Depressive y Symptoms (Mediator)

Depressive symptoms were measured using the CES-D scale (Center for Epidemiological Studies-Depression Scale) [29], translated and validated by Chen et al. [30]. This 20-item instrument uses a 4-point response format (0 = less than one day to 3 = five to seven days) for items such as “I feel lonely.” Higher mean scores indicate more severe symptoms. Cronbach’s alpha was 0.88 at Wave 1 and 0.84 at Wave 2.

#### 2.3.3. Teacher–Student Relationships (Moderator)

Teacher–student relations were assessed using the Teacher–Student Relations Subscale of the Chinese Version of the Delaware School Climate Scale-Student [31]. This subscale, adapted from the Delaware School Climate Scale [32], comprises 4 items (e.g., “Teachers care about their students”). Each item was scored on a 4-point scale ranging from 1, strongly disagree, to 4, strongly agree. The analysis was based on the average scores, with higher scores indicating a more positive relationship between teachers and students. Cronbach’s alpha was 0.92 at Wave 2.

#### 2.3.4. Peer Bullying Victimization (Dependent Variable)

Peer bullying victimization was evaluated using a four-item scale derived from the Panel Study of Income Dynamics, Child Development Supplement III [33], which has been previously utilized among Chinese adolescents [34]. The scale consists of four items, such as “I was blamed or verbally abused by peers” rated on a 5-point scale ranging from 0 (not once in the past month) to 4 (every day). Higher average scores across these items indicate a higher level of peer bullying victimization. Cronbach’s alpha was 0.73 at Wave 1 and 0.71 at Wave 2.

### 2.4. Statistical Analyses

First, prior to the main analyses, the data were screened for missing values, outliers, and entry errors. The proportion of missing values for each variable was less than 1%. Because the variables were not normally distributed, missing values were imputed using the median.

Second, descriptive statistics and bivariate correlation matrices for all study variables were computed using SPSS version 27.0. The PROCESS macro (Model 4) was used to examine the mediating role of depressive symptoms in the relationship between interparental conflict and peer bullying victimization. The PROCESS macro (Model 58) was used to test whether teacher–student relationships moderated the indirect effect of interparental conflict on peer bullying victimization—i.e., by moderating both the path from interparental conflict to depressive symptoms (first stage) and the path from depressive symptoms to peer bullying victimization (second stage).

Third, we controlled for gender and age by entering them as covariates into all analytical models. CIs of 95% for indirect effects were estimated via bias-corrected percentile bootstrap based on 5000 resamples. Any effect whose 95% CI did not span zero was deemed statistically meaningful. Two-tailed significance tests were applied throughout the analyses, with the alpha threshold fixed at *p* < 0.05.

Before conducting the main analyses, we examined regression assumptions. Multicollinearity was assessed using variance inflation factors, which ranged from 1.01 to 1.56, all below the threshold of 5, indicating no serious multicollinearity. Durbin–Watson values were close to 2 (ranging from 1.93 to 2.17), supporting the independence of residuals. Additionally, Cook’s distance values ranged from 0.000 to 0.687, all below 1, and leverage values were within acceptable limits, indicating that no individual case exerted undue influence on the regression results.

## 3. Results

### 3.1. Sample Characteristics

The final valid sample consisted of 759 adolescents. Among them, boys accounted for 49.8% and girls for 50.2%; seventh graders made up 50.6% and eighth graders 49.4%. Only-child families accounted for 9.0%, two-child families for 61.4%, and three-child and above families for 29.6%. In terms of household registration and residence, 26.1% of the participants were from rural areas, 44.4% from townships, and 29.5% from urban areas. In terms of paternal educational level, primary school education accounted for 16.5%, junior high school education for 56.3%, senior high school/secondary vocational education for 19.6%, and college education and above for 7.6%. In terms of maternal educational level, primary school education accounted for 20.7%, junior high school education for 55.9%, senior high school/secondary vocational education for 16.3%, and college education and above for 7.1%. At Wave 1, participants had a mean age of 13.56 years, ranging from 12 to 16 years. See Table 1.

### 3.2. Preliminary Analyses

Descriptive statistics (means and standard deviations) as well as correlation coefficients for all research variables are displayed in Table 2. Interparental conflict at Wave 1 was positively associated with peer bullying victimization at Wave 1 and Wave 2. Additionally, interparental conflict at Wave 1 was positively associated with depressive symptoms at Wave 1 and Wave 2. Depressive symptoms at Wave 1 and Wave 2 were positively associated with peer bullying victimization at Wave 1 and Wave 2. Furthermore, teacher–student relationships at Wave 2 were negatively associated with peer bullying victimization at Wave 1 and Wave 2.

### 3.3. Mediation Effect of Depressive Symptoms

**Figure 1:**
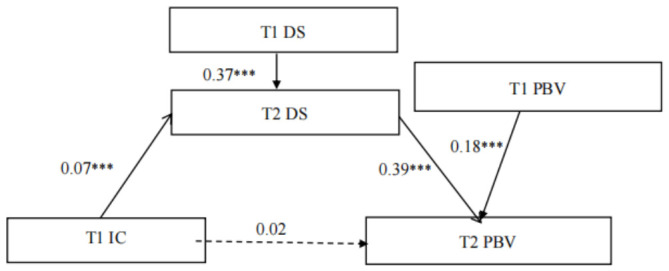
Mediating effect of depressive symptoms on the relationship between interparental conflict and peer bullying victimization. IC = interparental conflict; DS = depressive symptoms; PBV = peer bullying victimization. *** *p* < 0.001.

Figure 1 presents the results of the mediation model, controlling for gender, age, depressive symptoms, and peer bullying victimization at Wave 1; interparental conflict at Wave 1 positively predicted depressive symptoms at Wave 2 (*b* = 0.07, *SE* = 0.02, *β* = 0.13, *p* < 0.001), which in turn positively predicted peer bullying victimization at Wave 2 (*b* = 0.39, *SE* = 0.04, *β* = 0.40, *p* < 0.001). The bias-corrected percentile bootstrap method (5000 resamples) revealed that depressive symptoms at Wave 2 significantly mediated the relationship between interparental conflict at Wave 1 and peer bullying victimization at Wave 2 (indirect effect = 0.03, *SE* = 0.01, 95% CI = [0.01, 0.05]); completely standardized indirect effect = 0.05, 95% CI = [0.02, 0.08]). Details are shown in Table 3.

### 3.4. Moderated Mediation

**Figure 2:**
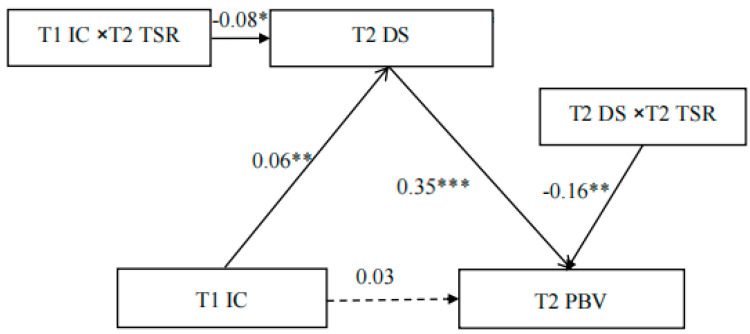
Moderating effect of teacher–student relationships on the indirect path from interparental conflict to peer bullying victimization via depressive symptoms. IC = interparental conflict; DS = depressive symptoms; PBV = peer bullying victimization; TSR = teacher–student relationship. * *p* < 0.05. ** *p* < 0.01. *** *p* < 0.001.

**Figure 3:**
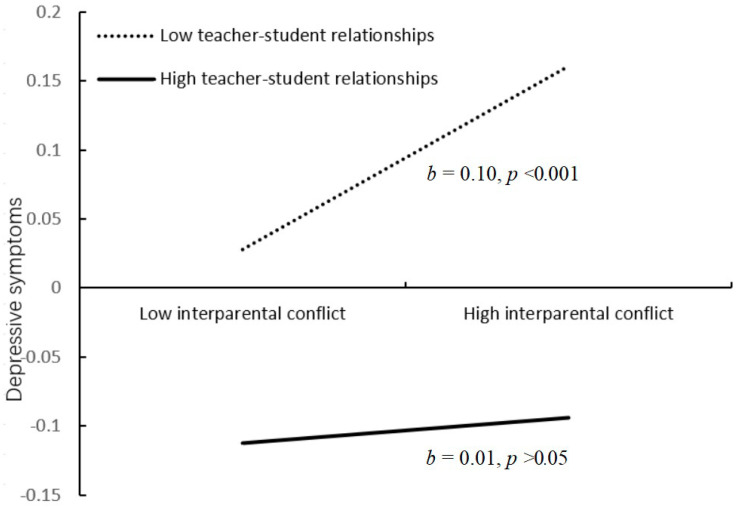
Moderating effect of teacher–student relationships on the association between interparental conflict and depressive symptoms.

**Figure 4:**
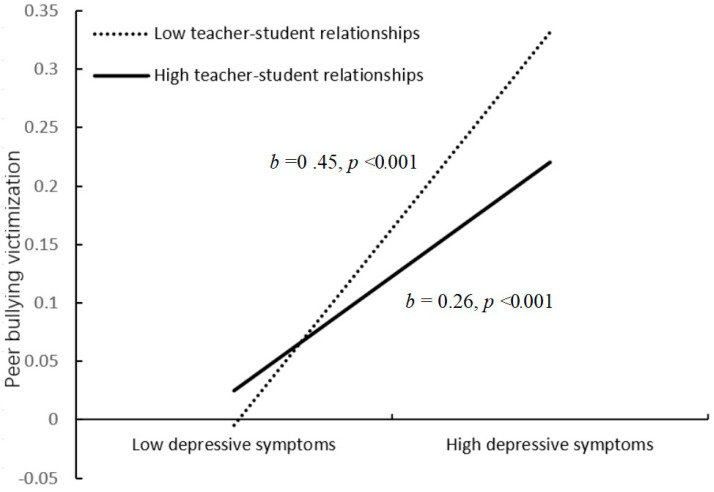
Moderating effect of teacher–student relationships on the association between depressive symptoms and peer bullying victimization.

Figure 2 illustrates the findings from the moderated mediation analysis. After controlling for gender, age, depressive symptoms, and peer bullying victimization at Wave 1, teacher–student relationships at Wave 2 moderated the mediation process. Specifically, teacher–student relationships at Wave 2 significantly moderated the path from interparental conflict at Wave 1 to depressive symptoms at Wave 2 (*b* = −0.08, *SE* = 0.03, *β* = −0.08, *p* < 0.05) and the path from depressive symptoms at Wave 2 to peer bullying victimization at Wave 2 (*b* = −0.16, *SE* = 0.05, *β* = −0.10, *p* < 0.01). The overall moderated mediation model explained 34% of the variance in depressive symptoms (R^2^ = 0.34) and 21% of the variance in peer bullying victimization (R^2^ = 0.21). We performed simple slope analyses to further elucidate the findings, with teacher–student relationships serving as the moderator. As illustrated in Figure 3, among adolescents who reported low teacher–student relationships at Wave 2, the link between interparental conflict at Wave 1 and depressive symptoms at Wave 2 was significant (*b* = 0.10, *SE* = 0.03, *β* = 0.18, *p* < 0.001). However, for those who reported high teacher–student relationships at Wave 2, this association was not significant (*b* = 0.01, *SE* = 0.03, *β* = 0.02, *p* > 0.05). Similarly, as illustrated in Figure 4, among adolescents who reported low teacher–student relationships at Wave 2, the link between depressive symptoms at Wave 2 and peer bullying victimization at Wave 2 was significant (*b* = 0.45, *SE* = 0.05, *β* = 0.45, *p* < 0.001). However, for those who reported high teacher–student relationships at Wave 2, this relation was weaker, although still statistically significant (*b* = 0.26, *SE* = 0.06, *β* = 0.26, *p* < 0.001). Details are shown in Table 4. 

## 4. Discussion

This study examined the relationship between interparental conflict and peer bullying victimization among Chinese adolescents, focusing on the mediating role of depressive symptoms and the moderating role of teacher–student relationships. As hypothesized, interparental conflict was positively associated with adolescent peer bullying victimization six months later, and depressive symptoms mediated this longitudinal association. More importantly, teacher–student relationships significantly moderated this indirect pathway: positive teacher–student relationships not only attenuated the association between interparental conflict and depressive symptoms to non-significance, but also weakened the link between depressive symptoms and peer bullying victimization. These findings extend prior research, revealing that depressive symptoms serve as a key mediating pathway linking family conflict to school victimization, and supportive teacher–student relationships may function as a protective factor.

Consistent with Hypothesis 1, the longitudinal data showed that interparental conflict was positively associated with peer bullying victimization among adolescents six months later. This result aligns with previous research [6,9], further suggesting that interparental conflict may be an important risk-related factor. One possible explanation, drawn from the bioecological model of human development [11], is that negative proximal processes within the family (e.g., interparental conflict) may impair adolescents’ psychosocial functioning (e.g., hindering social skills development), leading to maladaptive peer interactions and thus being associated with an elevated risk of peer bullying victimization. Overall, this longitudinal study provided evidence for the significant association between interparental conflict and peer bullying victimization among adolescents.

It should be noted that although interparental conflict was significantly positively correlated with peer bullying victimization in this study, the longitudinal correlation coefficient was only r = 0.14, indicating a small effect size. This suggests that the predictive effect of interparental conflict on peer bullying victimization is diluted by other external variables. Adolescent development is influenced by multiple factors at both the individual and ecological environmental levels. For example, peer support networks may provide protective resources that buffer the negative effects of interparental conflict; school anti-bullying policies may reduce peer bullying victimization; and adolescent personality traits (e.g., psychological resilience) may weaken the adverse impact of interparental conflict. Therefore, statistical significance does not equate to high practical significance. In terms of intervention, efforts should not rely solely on the family context but should also involve a collaborative approach that includes schools and peer groups.

Consistent with Hypothesis 2, this study found that depressive symptoms significantly mediated the relationship between interparental conflict and adolescent peer bullying victimization. Specifically, interparental conflict was associated with an increased risk of peer bullying victimization among adolescents through exacerbating their depressive symptoms. This finding highlights the potential pathway from interparental conflict to adolescents’ emotional symptoms and how this pathway extends to their peer environment. One possible explanation is that adolescents exposed to interparental conflict experience threats to their emotional security, which can lead to the development of depressive symptoms. Depressive symptoms are often accompanied by social isolation [35], which makes adolescents more likely to become targets of bullying in peer interactions. This mediating pathway not only reveals how interparental conflict could be related to peer bullying victimization through its association with adolescents’ emotional symptoms but also supports both the emotional security theory [12] and the symptom-driven model [14,15,16].

Consistent with Hypothesis 3, teacher–student relationships moderated the indirect pathway linking interparental conflict to peer bullying victimization through depressive symptoms, and the buffering effect operated at both stages of the mediation process. Specifically, as a protective resource, positive teacher–student relationships attenuated the association between interparental conflict and depressive symptoms, and weakened the correlation between depressive symptoms and peer bullying victimization. One possible explanation is that supportive teacher–student relationships provide adolescents with emotional security and a sense of belonging at school, counteracting the emotional insecurity triggered by interparental conflict, thereby making them less likely to develop depressive symptoms. Furthermore, positive teacher–student relationships can reduce social withdrawal, which in turn lowers their risk of becoming targets of bullying. These findings support the stress-buffering model [20,21] and align with previous studies [22,23], further validating that the protective role of positive teacher–student relationships can protect adolescents against adverse developmental outcomes.

In addition, the number of children in the family is an important potential confounding variable. On the one hand, raising multiple children requires the division of financial and time resources, which tends to exacerbate interparental conflict. On the other hand, sibling relationships exert dual effects: siblings can provide companionship and emotional support to mitigate the adverse consequences of interparental conflict, while conflicts between siblings may bring additional stressors and amplify negative outcomes. Existing studies have verified that sibling relationships moderate the association between stressful life events and internalizing problems [36], and sibling bullying victimization can predict peer bullying victimization among adolescents [37]. Therefore, the number of siblings is likely to moderate the associations among interparental conflict, depressive symptoms and peer bullying victimization.

This study had several limitations. First, this study relied exclusively on adolescent self-report data, which may introduce common method bias and social desirability bias. Although the two-wave data collection with a six-month interval may reduce this risk to some extent, common method variance remains a potential limitation. To improve the validity of the conclusions, future research should integrate multi-informant reports (e.g., from parents, teachers, or peers) to obtain more objective and comprehensive measurements. Second, although this study adopted a two-wave longitudinal design, the mediator (depressive symptoms) and the outcome variable (peer bullying victimization) were measured simultaneously at Wave 2. Accordingly, the temporal sequence of the mediator relative to the outcome variable cannot be confirmed. Future research may adopt a three-wave longitudinal design to more clearly clarify the temporal associations among variables. The theoretical rationale is that, according to the emotional security theory, interparental conflict serves as a distal family stressor that undermines adolescents’ emotional security, making depressive symptoms a key mechanism linking family adversity to peer difficulties. However, existing longitudinal studies have validated the reverse path: peer bullying victimization can also predict subsequent depressive symptoms [38]. A bidirectional association may exist between depressive symptoms and peer bullying victimization, and the present study cannot rule out the possibility of the reverse effect. Future research may adopt a cross-lagged panel model to simultaneously test both paths. Fourth, the sample consisted only of Chinese students, which limits the generalizability of the findings to other cultural contexts. In the collectivist cultural context of China, teacher–student relationships may have a stronger protective effect, whereas in individualistic cultures, this buffering effect may be relatively weaker. Therefore, future cross-cultural replication studies are needed to examine the generalizability of the present findings. Fifth, this study examined the moderating role of teacher–student relationships. Future research should explore other protective factors within the school ecosystem, such as peer support [39], to more comprehensively reveal the complex mechanisms that buffer adolescents against the risk of peer bullying victimization. Sixth, the proportion of multi-child families in the sample is high, which limits the external generalizability of the research conclusions. Follow-up studies may quantitatively examine the moderating effect of sibling number in this chained pathway.

Implications for practice: First, interparental conflict is an important risk factor for adolescent peer bullying victimization. Therefore, schools should strengthen home–school collaboration and enhance teachers’ understanding of students’ family backgrounds to facilitate early identification of at-risk adolescents. Second, the mediating role of depressive symptoms reveals a key mechanism linking family conflict to peer bullying victimization. Thus, attention should be paid to adolescents showing depressive symptoms. Schools may implement early interventions targeting depressive symptoms (e.g., emotion regulation training, cognitive-behavioral groups, counseling services) to alleviate adolescents’ internalizing distress and lower their risk of peer bullying victimization. Third, fostering positive teacher–student relationships is an effective strategy to buffer the indirect effect of interparental conflict on peer bullying victimization. Accordingly, schools should strengthen teacher training to help teachers build warm, supportive, and trusting relationships with students (e.g., by showing empathy, providing academic encouragement, and maintaining open communication), thereby enhancing the protective role of teacher–student relationships.

## 5. Conclusions

The findings indicate that interparental conflict was positively associated with adolescent peer bullying victimization six months later, and depressive symptoms mediated this longitudinal association. Importantly, positive teacher–student relationships exerted a buffering effect across two key stages: they attenuated the association between interparental conflict and depressive symptoms to non-significance, and weakened the link between depressive symptoms and peer bullying victimization.

From a practical perspective, the mediating effect of depressive symptoms suggests that early identification and intervention targeting adolescents’ depressive symptoms are necessary. Meanwhile, the buffering effect of teacher–student relationships indicates that supportive interpersonal connections at school can alleviate the negative impacts stemming from family-level risks.

## Figures and Tables

**Table 1:** Demographic characteristics of participants.

Variable	Item	*n* (%) or Mean ± SD
Gender	Male	378 (49.8)
	Female	381 (50.2)
Age		13.56 ± 0.68 (range 12–16)
Grade	Grade 7	384 (50.6)
	Grade 8	375 (49.4)
Sibling status	Only child	68 (9.0)
	Two-child family	466 (61.4)
	Three-child family	225 (29.6)
Paternal educational level	Primary school	125 (16.5)
	Junior high school	427 (56.3)
	Senior high school	149 (19.6)
	College and above	58 (7.6)
Maternal educational level	Primary school	157 (20.7)
	Junior high school	424 (55.9)
	Senior high school	124 (16.3)
	College and above	54 (7.1)
Residence	Rural areas	198 (26.1)
	Townships	337 (44.4)
	Urban areas	224 (29.5)

**Table 2:** Means, standard deviations, and correlations for all variables.

Variable	1	2	3	4	5	6
1. IC at Wave 1	1.00					
2. DS at Wave 1	0.35 ***	1.00				
3. DS at Wave 2	0.29 ***	0.51 ***	1.00			
4. PBV at Wave 1	0.24 ***	0.36 ***	0.21 ***	1.00		
5. PBV at Wave 2	0.14 ***	0.10 **	0.35 ***	0.28 ***	1.00	
6. TSR at Wave 2	−0.16 ***	−0.19 ***	−0.36 ***	−0.18 ***	−0.21 ***	1.00
Mean	2.02	0.71	0.57	0.32	0.16	3.24
SD	0.64	0.46	0.38	0.52	0.37	0.59

Note: IC = interparental conflict; DS = depressive symptoms; PBV = peer bullying victimization; TSR = teacher–student relationship. ** *p* < 0.01. *** *p* < 0.001.

**Table 3:** Mediating effect of depressive symptoms on the relationship between interparental conflict and peer bullying victimization.

	Model 1 (T2 DS)				Model 2 (T2 PBV)			
	*b*	*SE*	*β*	*t*	*b*	*SE*	*β*	*t*
Gender	−0.01	0.02	−0.02	−0.49	0.04	0.02	0.06	1.72
Age	−0.03	0.02	−0.05	−1.51	0.01	0.02	0.01	0.37
T1 IC	0.07	0.02	0.13	3.73 ***	0.02	0.02	0.04	1.17
T1 DS	0.37	0.03	0.45	12.95 ***				
T1 PBV					0.18	0.03	0.26	7.34 ***
T2 DS					0.39	0.04	0.40	10.35 ***
R^2^, Δ R^2^	0.28, 0.02				0.20, 0.12			
df1, df2	5, 753				6, 752			
F	57.47 ***				31.55 ***			

Note: IC = interparental conflict; DS = depressive symptoms; PBV = peer bullying victimization. *** *p* < 0.001.

**Table 4:** Moderating effect of teacher–student relationships on the indirect path from interparental conflict to peer bullying victimization via depressive symptoms.

	Model 1 (T2 DS)				Model 2 (T2 PBV)			
	*b*	*SE*	*β*	*t*	*b*	*SE*	*β*	*t*
Gender	−0.01	0.02	−0.03	−0.52	0.04	0.02	0.11	1.74
Age	−0.02	0.02	−0.04	−1.34	0.01	0.02	0.01	0.30
T1 IC	0.06	0.02	0.10	3.12 **	0.03	0.02	0.04	1.24
T1 DS	0.36	0.03	0.43	12.73 ***				
T1 PBV					0.18	0.03	0.25	7.05 ***
T2 TSR	−0.17	0.02	−0.26	−8.58 ***	−0.03	0.02	−0.06	−1.57
T1 IC × T2 TSR	−0.08	0.03	−0.08	−2.39 *				
T2 DS					0.35	0.04	0.36	8.93 ***
T2 DS × T2 TSR					−0.16	0.05	−0.10	−2.88 **
R^2^, Δ R^2^	0.34, 0.08				0.21, 0.13			
df1, df2	7, 751				8, 750			
F	56.29 ***				25.41 ***			

Note: IC = interparental conflict; DS = depressive symptoms; PBV = peer bullying victimization; TSR = teacher–student relationship. * *p* < 0.05. ** *p* < 0.01. *** *p* < 0.001.

## Data Availability

The data presented in this study are available on request from the corresponding authors (the data are not publicly available due to privacy or ethical restrictions).

## Abbreviations

The following abbreviations are used in this manuscript:

IC	Interparental conflict
DS	Depressive symptoms
TSR	Teacher–student relationship
PBV	Peer bullying victimization
